# Permutation test for periodicity in short time series data

**DOI:** 10.1186/1471-2105-8-395

**Published:** 2007-10-17

**Authors:** Andrey A Ptitsyn, Sanjin Zvonic, Jeffrey M Gimble

**Affiliations:** 1 Center for Bioinformatics, Colorado State University Campus delivery 1682 Fort Collins, CO 80523, USA; 2 Stem Cell Laboratory, Louisiana State University System Pennington Biomedical Research Center, 6400 Perkins Rd., Baton Rouge, LA 70808, USA

In our paper reporting the permuted-time test for periodicity [1], the table in Figure 4 and the text of the Discussion should be modified as follows: The % of genes detected by Tu *et al*. [2] should be reported as 57.2% rather than 38.1% as currently reported; thus the difference between the methods should be reduced (to 84.7% vs. 57.2%). The discrepancy results from the fact that the absolute number of 3552 transcripts was identified from a smaller subset of pre-selected expression profiles corresponding to known yeast genes, while the Pt-test was applied to the entire set of probesets. We would like to thank two of the co-authors of the Tu *et al. *[2] publication, Drs. Rowicka and Kudlicki (UT-Southwestern), for bringing this to our attention.
